# Calreticulin and PDIA3, two markers of endoplasmic reticulum stress, are associated with metabolic alterations and insulin resistance in pediatric obesity: A pilot study

**DOI:** 10.3389/fendo.2022.1003919

**Published:** 2022-09-23

**Authors:** Valentina Antoniotti, Simonetta Bellone, Filipa Patricia Gonçalves Correia, Caterina Peri, Sabrina Tini, Roberta Ricotti, Valentina Mancioppi, Mara Gagliardi, Daniele Spadaccini, Marina Caputo, Marco Corazzari, Flavia Prodam

**Affiliations:** ^1^ Struttura Complessa a Direzione Universitaria (SCDU) of Pediatrics, Department of Health Sciences, University of Piemonte Orientale, Novara, Italy; ^2^ Department of Health Sciences, University of Piemonte Orientale, Novara, Italy; ^3^ Center for Translational Research on Autoimmune and Allergic Disease (CAAD) & Interdisciplinary Research Center of Autoimmune Diseases (IRCAD), University of Piemonte Orientale, Novara, Italy; ^4^ Endocrinology, Department of Translational Medicine, University of Piemonte Orientale, Novara, Italy

**Keywords:** obesity, ER stress, pediatrics, insulin, lipids

## Abstract

Our aim was to evaluate the markers of endoplasmic reticulum (ER) stress among children and adolescents with obesity in relation to metabolic alterations. Calreticulin (CALR) and PDIA3 circulating levels were assessed on 52 pediatric subjects—26 patients with obesity and 26 normal weight controls (4–18 years)—enrolled in a pilot study. Clinical and metabolic evaluations were performed (BMI-SDS, insulin, and glucose at fasting and during an oral glucose tolerance test, lipid profile, blood pressure), and metabolic syndrome was detected. PDIA3 was higher (*p* < 0.02) and CALR slightly higher in children with obesity than in controls. PDIA3 was related positively to the Tanner stages. Both PDIA3 and CALR were positively associated with insulin resistance, cholesterol, and triglycerides and the number of criteria identifying metabolic syndrome and negatively with fasting and post-challenge insulin sensitivity. Our preliminary findings suggest the existence of a link between ER stress and metabolic changes behind obesity complications even at the pediatric age. CALR and PDIA3 could be early markers of insulin resistance and dyslipidemia-related ER stress useful to stratify patients at high risk of further complications.

## 1 Introduction

Obesity is a growing public health issue due to its prevalence and numerous associated comorbidities in children and adolescents (1–4). Inflammation, oxidative stress, lipotoxicity, and endoplasmic reticulum (ER) stress are all identified as obesity triggers for its complications (5).

Some hypotheses have been proposed in order to clarify the mechanisms leading to proinflammatory response activation of visceral adipose tissue (VAT) (6), considering the influence of several mechanisms on adipose tissue, including the hypoxia-inducible factor (HIF) activation (7, 8), the production of reactive oxygen species (ROS) (9), the increased concentration of free fatty acids (FFAs) leading to lipotoxicity, and the role of proinflammatory cytokines (6). This lays the foundation for the development of complications such as insulin resistance and type 2 diabetes mellitus (T2DM) (10).

The excess of nutrients and the increased metabolism of FFAs, characteristics of obesity, can lead to the involvement of the endoplasmic reticulum (ER), since it plays a crucial role in controlling lipid metabolism by regulating lipid synthesis, modification, and secretion (11).

**Figure 1:**
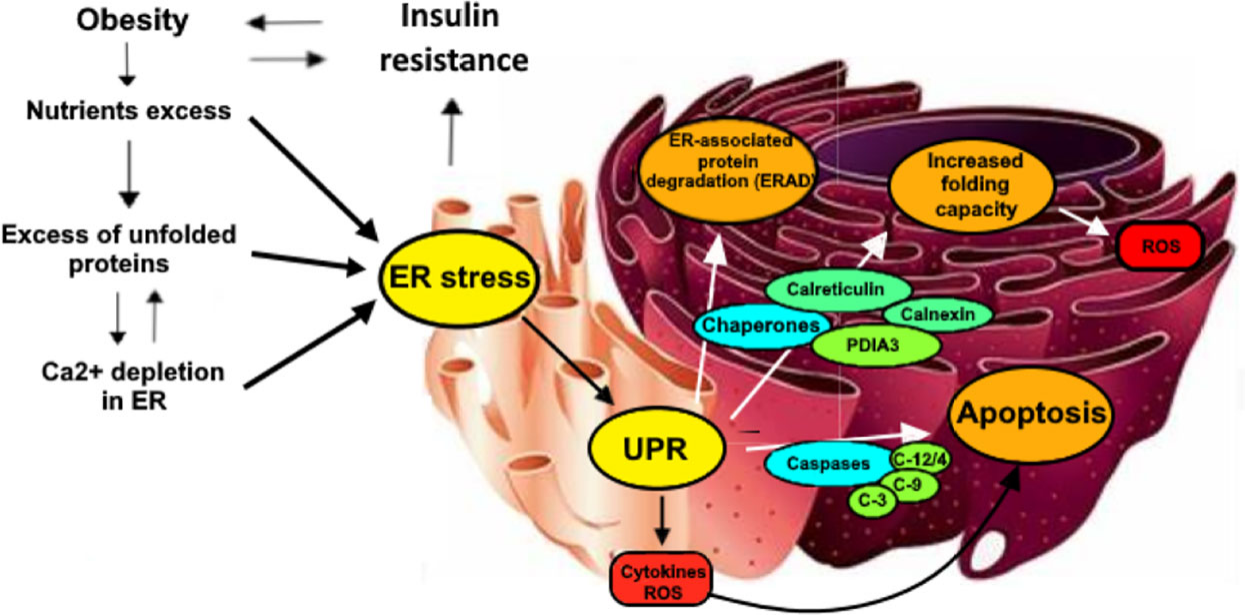
Some of the main mechanisms underlying the relationship between obesity, insulin resistance al URP-related chaperones activity. Nutrients intake, when is higher than cellular needs, causes an overproduction of unfolded proteins that require to be processed by ER. In these situations, ER activates multiple mechanisms to reduce stress sources. First, Insulin resistance is provoked by the cell to reduce the insulin-mediated protein transcription, lately being ad additional factor for the development of obesity. Secondly, ER activates UPR, increasing the activity of several enzymes such as caspases that are responsible of apoptotic processes in addition to ROS and inflammatory cytokines production. URP has also the role of increasing protein degradation (ERAD) and contemporarily of boosting folding capacity through oxidative protein folding, which is partially performed by the activation of the PDIA3/Calreticulin/Calnexin chaperones complex..

When the ER is subjected to excessive workload, it enters a stress situation (ER stress); thus, to protect cellular functionality, unfolded protein response (UPR) is activated, with the aim of solving ER stress by acting on cellular metabolism, through a process of retro-translocation in the cytoplasm, the addition of ubiquitin, and demolition by the proteasome (11). However, when the protein load is excessive and the ER stress is very high, the UPR leads to the activation of cellular death mechanisms. This situation can be observed in obesity when several chronic conditions such as hyperglycemia, hyperinsulinemia, and elevation of FFA plasma levels and proinflammatory cytokines promote the activation of the cell death pathway mediated by ER stress (12). Indeed, an increase in the expression of proteins involved in cytoskeletal remodeling, inflammation, cellular senescence, oxidative stress, and ER stress can be observed particularly in those patients showing signs of metabolic decompensation, such as insulin resistance or T2DM (13–16). It seems that some of the proteins involved in the abovementioned processes may represent valid markers of “progression” of the disease and could therefore contribute to the identification of complications at an early stage. Among these, calreticulin (CALR) and PDIA3, two important proteins located in the ER and involved in cellular response to ER stress, whose increase is part of the attempt of cells to cope with some of the deleterious effects of cell stress, such as protein misfolding and ROS damage, are worthy of a more in-depth analysis (13–18) (**Figure 1**).

CALR was initially described as an ER protein, regulating calcium homeostasis and the folding of glycoproteins (17). Today, CALR is recognized as a chaperone with various functions; it is involved in the regulation of cell proliferation processes, phagocytosis, apoptosis, adhesion, and adaptive and innate immune responses. Acting synergistically with calnexin (CNX), another chaperone of the ER, CALR becomes part of the quality control mechanism that monitors the state of glycosylation and three-dimensional arrangement of proteins transiting the ER (17, 19).

CALR functions become fundamental to resolve the accumulation of proteins within the ER and ensure a return to cellular homeostasis. In these conditions and following the activation of the UPR, there is an increase in the synthesis of this protein, and IRE-1 (an enzyme recruited as a first response) mediates the processing and activation of XBP-1, a transcription factor that acts on the genes of several chaperones, including CALR and CNX themselves (19, 20). It is through this mechanism that an increased CALR expression is observed in tissues subjected to strong cellular stress, just like in the case of VAT, subcutaneous adipose tissue (SAT), and the liver of obese subjects, in which CALR is found in high concentrations (14, 21).

*PDIA3* is among the genes whose transcription is promoted by XBP-1 (19, 20). PDIA3 is present mainly but not exclusively in the ER, where its main function is to ensure and mediate the formation of the correct intramolecular disulfide bonds in nascent proteins. Indeed, PDIA3 is a stress response protein aimed at avoiding and solving situations of cellular stress (22), and its expression is increased in these cases, especially following protein accumulation that results in ER stress and UPR (14–16, 21).

This protein has four domains, with an active site that provides its oxidation-reductive property, while the C-terminal possibly enables protein retention in the ER and could act as a nuclear localization sequence and a portion responsible for the interaction and binding with CALR and CNX, contributing to normal folding and proper protein glycosylation interaction (22). PDIA3 is also involved in various functions in other cellular compartments, with studies supporting its involvement in signal transduction from the cell surface, the regulation of nuclear processes such as DNA repair, and the promotion of phagocytosis and autophagy (18).

Based on the above, we aimed to assess if an association among CALR, PDIA3, and metabolic risk factors in pediatric obesity exists since ER stress proteins could be precocious markers of metabolic impairment and no data exist in this age on these molecules.

## 2 Materials and methods

### 2.1 Population and clinical parameters

Fifty-two subjects aged between 4 and 18 were enrolled. Twenty-six case subjects were recruited in a pilot study from patients attending the Division of Pediatric Endocrinology of our hospital. As a control group, 26 normal weight age-matched subjects were enrolled. The control group consisted of outpatients of our hospital, joining the project “The prevention game begins as a child” (CE 95/12). The inclusion criteria for patients were the presence of obesity according to the International Obesity Task Force curves (IOTF) (23). For both groups, we excluded subjects with cardiorespiratory diseases, endocrine diseases (hypo- or hyperthyroidism, growth hormone deficiency, type 1 diabetes, adrenal insufficiency, hypercortisolism), chronic diseases, and genetic causes of obesity, as well as those under prescription drugs that could alter glucose and/or lipid metabolism.

All patients were subjected to clinical assessment, including Tanner criteria for pubertal development, defining puberty at stages 2–5 (24). Weight, height, and waist circumference were measured by the medical staff, and BMI and BMI *Z*-score were calculated. Patients and controls were categorized into weight classes according to the 2012 IOTF (23).

Blood pressure was measured and stratified by gender and age, as suggested by the National High Blood Pressure Education Program (NHBPEP) Working Group of the American Academy of Pediatrics (AAP) (25).

### 2.2 Biochemical evaluation

Blood samples were collected at fasting in the morning. We evaluated glucose and insulin, total cholesterol, HDL, triglycerides, PDIA3, and CALR. All patients were subjected to an oral glucose tolerance test (OGTT), evaluating glucose and insulin levels every 30 min for 120 min (26). From the OGTT, the indexes regarding insulin resistance (HOMA-IR) and insulin sensitivity (QUICKI and Matsuda index, ISI) as well as the insulinogenic index were calculated. The formulas are reported in our previous papers (27, 28).

LDL levels were obtained from Friedwald’s formula, calculated for subjects with triglyceride levels below 150 mg/dl.

Patients were also evaluated for the presence of cardiovascular risk factors considered in the definition of metabolic syndrome using the limit values of the modified criteria of the National Cholesterol Education Program (NCEP) and the Adult Treatment Panel (ATP) III (>90 percentile triglycerides by sex and age; <10 percentile HDL by sex and age; IFG/IGT) (26).

### 2.3 CALR and PDIA3 analysis

CALR or PDIA3 concentration was evaluated in plasma samples using the Human CALR or PDIA3 ELISA kit [Elabsicence (Houston, Texas, USA)]. Briefly, a 100-μl dilution consisting of standard, blank, and sample was placed into the appropriate wells (in duplicate). Samples were incubated for 90 min at 37°C. Liquid was decanted from each well, and 100 μl of Biotinylated Detection Ab working solution was added to each well immediately. The samples were incubated for 1 h at 37°C. The liquid was decanted and 350 μl of wash buffer was added to each well and removed after 1 min (repeated three times). Then, 100 μl of HRP conjugate working solution was added to each well, and the plate was incubated for 30 min at 37°C. The liquid was removed and each was washed three times as reported above. Then, 90 μl of substrate reagent was added to each well, and the samples were incubated for about 15 min at 37°C (protected from light). Fifty microliters of stop solution was added to each well, and optical density (OD value) was evaluated with a microplate reader set to 450 nm [Spark multimode microplate reader; Tecan (Seestrasse, Switzerland)].

CALR or PDIA3 concentration in each sample was calculated through the standard curve, obtained by serial dilution of a CALR or PDIA3 stock solution of 10 ng/ml. The sensitivity of the kit was estimated to be 0.10 ng/ml, the analytical range was between 0.16 and 10 ng/ml, and the coefficient of variation was <10%.

### 2.4 Statistical analysis

Due to the small sample of patients, a non-parametric approach was used. Data were expressed as median (IQR) or percentage (%). Differences between the two groups were analyzed using the Mann–Whitney *U* test. Correlation analysis was performed using Spearman’s *r*.

Statistical significance was set at *p <*0.05. The statistical analysis was performed using SPSS for Windows V.26.0 (SPSS Inc., Chicago, IL, USA).

## 3 Results

This pilot study was carried out involving 52 subjects (26 healthy subjects and 26 with obesity) matched for sex and pubertal stage. Six of them were excluded because of incomplete clinical information. The clinical data are reported in **Table 1**. Of the children with obesity, 65.2% were obese and 34.8% were morbidly obese.

**Table 1:** Clinical characteristics of the population.

Variable		Obese	Normal weight	*p*-value
Gender	Male	12 (52.2%)	5 (40.3%)	
	Female	11 (47.8%)	18 (59.7%)	
Puberty	Prepubertal	8 (34.8%)	4 (17.4%)	
	Pubertal	15 (65.2%)	19 (82.6%)	
Age (years)		11.4 (8.8; 13.4)	12.6 (9.9; 13.2)	
Height (cm)		155.3 (137.8; 161)	152.5 (145; 163.1)	
Weight (kg)		69.1 (52; 81.5)	40 (34; 48.2)	*p* < 0.001
BMI (kg/m^2^)		28.4 (26.1; 32.4)	17 (15.7; 18.2)	*p* < 0.001
BMI *Z*-score (kg/m^2^)		2.03 (1.9; 2.5)	−0.94 (−1.32; −0.40)	*p* < 0.001
Waist (cm)		92 (78; 99)	69 (63.5; 72.5)	*p* < 0.001
Waist/heigh (cm)		0.58 (0.54; 0.61)	0.38 (0.01; 0.44)	*p* < 0.001

Data are expressed as median (IQR) or percentage (%).

BMI, body mass index.

Children with obesity had a higher level of PDIA3 (0.212, 0.187–0.465 ng/ml, *p* < 0.05) than normal weight patients (0.188, 0.167–0.222 ng/ml), corrected by sex and age. PDIA3 significance was lost when correcting also for BMI and pubertal stage. CALR levels, on the other hand, were comparable between normal weight patients (0.230, 0.206–0.273 ng/ml) and those with obesity (0.233, 0.145–0.422 ng/ml).

### 3.1 Metabolic features

Glucose and insulin levels at 0 min (*p* < 0.01) and 120 min (*p* < 0.001) and HOMA-IR (*p* < 0.001) were higher, while HDL (*p* < 0.001), QUICKI (*p* < 0.001), and ISI (*p* < 0.001) were lower in obese subjects compared to normal weight subjects. The data are reported in **Table 2**.

**Table 2:** Metabolic and biochemical characteristics of obese and normal weight individuals.

	Obese (*N* = 23)	Normal weight (*N* = 23)	*p*-value
Systolic blood pressure (mmHg)	123 (112; 132)	120 (110; 125)	
Systolic blood pressure (mmHg)	80 (70; 86)	80 (70; 80)	
Total cholesterol (mg/dl)	141 (121; 158)	142 (127; 164.5)	
HDL (mg/dl)	40 (36; 54)	60 (49; 65)	*p* < 0.001
LDL (mg/dl)	82 (63; 100)	73 (61; 96)	
Non-HDL-c (mg/dl)	97 (78; 120)	84 (72; 105.5)	
TG (mg/dl)	63 (38; 91)	44 (40.5; 54)	*p* < 0.04
AST (mg/dl)	24 (19; 28)	25 (23.5; 29)	
ALT (mg/dl)	21 (18; 30)	19 (17.5; 21.5)	
Glucose at 0 min (mg/dl)	89 (84; 95)	85 (75; 89.5)	*p* < 0.01
Glucose at 120 min (mg/dl)	117 (106; 123)	91 (86.5; 108.5)	*p* < 0.001
Insulin at 0 min (UI/ml)	16.8 (9.3; 24.6)	9.5 (6.3; 11.3)	*p* < 0.001
Insulin at 120 min (UI/ml)	70.7 (43.7; 135.5)	24.5 (17.4; 37.4)	*p* < 0.001
HOMA-IR	3.7 (2.2; 5.8)	2.0 (1.3; 2.3)	*p* < 0.001
QUICKI	0.31 (0.29; 0.34)	0.34 (0.33; 0.36)	*p* < 0.001
ISI	3.42 (1.87; 4.41)	6.14 (5.53; 8.32)	*p* < 0.001
Insulinogenic index	1.99 (0.89; 2.93)	1.32 (1.19; 1.91)	
CALR (ng/ml)	0.233 (0.145; 0.422)	0.230 (0.206; 0.273)	
PDIA3 (ng/ml)	0.212 (0.187; 0.465)	0.188 (0.167; 0.222)	*p* < 0.05

Descriptive characteristics are expressed as median (IQR).

HDL, high-density lipoprotein; LDL, low-density lipoprotein; TG, triglyceride; AST, aspartate aminotransferase; ALT, alanine aminotransferase; HOMA-IR, insulin resistance index; QUICKI, quantitative insulin-sensitivity check index; ISI, insulin sensitivity index.

### 3.2 Correlation analysis

#### 3.2.1 CALR

CALR was positively correlated with systolic (*r* = 0.329; *p* < 0.05) and diastolic blood pressure (*r* = 0.374; *p* < 0.05), acanthosis score (*r* = 0.684; *p* < 0.001), non-HDL cholesterol (*r* = 0.347; *p* < 0.03), triglycerides (*r* = 0.488; *p* < 0.003), number of criteria identifying metabolic syndrome (*r* = 0.705; *p* < 0.001), fasting insulin (*r* = 0.415; *p* < 0.005), HOMA-IR (*r* = 0.368; *p* < 0.005), and insulinogenic index (*r* = 0.292; *p* < 0.05). CALR was, instead, negatively correlated with HDL (*r* = −0.390; *p* < 0.01), QUICKI (*r* = −0.368; *p* < 0.01), and ISI (*r* = −0.421; *p* < 0.02) (**Table 3**).

**Table 3:** Significant correlation of CALR and PDIA3.

	CALR	Spearman’s *r*	*p*-value	PDIA3	Spearman’s *r*	*p*-value
Weight (kg)				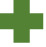	0.325	0.001
BMI (kg/m^2^)				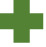	0.303	0.03
BMI *Z*-score (kg/m^2^)				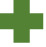	0.378	0.008
Waist circumference (cm)				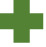	0.292	0.05
Diastolic blood pressure (mmHg)	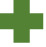	0.329	0.05			
Systolic blood pressure (mmHg)	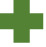	0.317	0.05			
Tanner stages				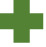	0.328	0.05
Acanthosis	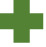	0.684	0.001	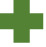	0.342	0.05
LDL (mg/dl)				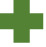	0.305	0.05
HDL (mg/dl)	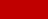	−0.390	0.01	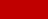	−0.433	0.008
Non-HDL cholesterol (mg/dl)	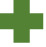	0.347	0.03	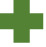	0.438	0.008
Triglycerides (mg/dL)	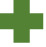	0.488	0.003	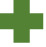	0.664	0.001
Number of criteria identifying metabolic syndrome	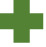	0.705	0.001	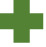	0.485	0.001
Fasting insulin (UI/ml)	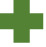	0.415	0.005	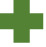	0.296	0.02
HOMA-IR	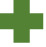	0.368	0.01	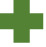	0.273	0.06
Insulinogenic index	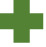	0.292	0.05			
QUICKI	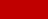	−0.368	0.01	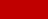	−0.283	0.06
ISI	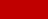	−0.421	0.02			

BMI, body mass index; HOMA-IR, insulin resistance index; QUICKI, quantitative insulin-sensitivity check index; ISI, insulin sensitivity index; 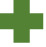, positive correlation; 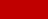, negative correlation.

#### 3.2.2 PDIA3

As shown in **Table 3**, PDIA3 levels were positively correlated with weight (*r* = 0.325; *p* < 0.001), BMI (*r* = 0.303; *p* < 0.03) and BMI *Z*-score (*r* = 0.378, *p* < 0.008), waist circumference (*r* = 0.292; *p* < 0.05), Tanner stages (*r* = 0.328; *p* < 0.05), acanthosis score (*r* = 0.342; *p* < 0.05), number of criteria identifying metabolic syndrome (*r* = 0.485; *p* < 0.001), LDL (*r* = 0.305; *p* < 0.05), non-HDL cholesterol (*r* = 0.438; *p* < 0.008), triglycerides (*r* = 0.664; *p* < 0.001), fasting insulin (*r* = 0.296; *p* < 0.02), and HOMA-IR (*r* = 0.273; *p* < 0.06). PDIA3 was found to be only inversely correlated with HDL (*r* = −0.433; *p* < 0.008) and QUICKI (*r* = −0.283; *p* < 0.06).

## 4 Discussion

Obesity is associated with several comorbidities, all related to the establishment of chronic inflammation, oxidative stress, lipotoxicity, and endoplasmic reticulum (ER) stress. Our preliminary data suggest that CALR and PDIA3, two key molecules of ER stress, could be related to insulin resistance and altered lipid profile in pediatric obesity.

One of the mechanisms involved in the development of obesity complications is ER stress, triggered by an overload of lipids and proteins among other mechanisms. ER stress markers have been found elevated in the visceral adipose tissue of adult patients with obesity, and it seems that these proteins could be markers also in their circulating form. Two of the most studied molecules are CALR and PDIA3, starting from their relation with cancer (18, 29–32). The markers of ER stress are poorly investigated especially at a young age. Therefore, we aimed to explore their modulation in children and adolescents with obesity.

First, preliminary data from our study showed higher circulating levels of PDIA3, but not CALR, in obese pediatric subjects compared to those with normal weight. The correlation of PDIA3 with BMI, weight, and waist circumference can be explained by the release of ER stress markers in the adipose tissue, increasing simultaneously with the increased number of adipocytes. PDIA3 is also an important regulator of postnatal bone and muscle growth, particularly during the pubertal growth spurt (33), possibly explaining also the correlation of PDIA3 with the Tanner stage. However, because our population is mainly in the mid of puberty, the strength of the association could be influenced by this age and puberty distribution.

Second, in our cohort, we observed associations between circulating levels of both markers and cardiometabolic risk factors. PDIA3 was directly correlated with a high concentration of LDL cholesterol, and both PDIA3 and CALR were directly correlated with non-HDL cholesterol and triglycerides and inversely correlated with low HDL cholesterol levels. Our results are in line with recent literature, reporting an increased lipid turnover as a cause of ER malfunction (12, 34). Furthermore, both molecules were involved in cholesterol assembly and turnover (35, 36).

PDIA3 and CALR were also correlated directly with insulin levels and HOMA-IR and inversely with ISI and QUICKI, surrogate indexes of insulin sensitivity. Also, both markers were found to be related with acanthosis nigricans, which is one of the clinical manifestations of insulin resistance, due to the presence of keratinocytes and fibroblasts able to respond to insulin through the expression of IGF1-R (37, 38). Literature studies confirm how ER stress is directly involved in the development of insulin resistance during obesity such as in a pediatric population (39–41). Our results strengthen these findings and underline how insulin resistance and ER stress are strictly related in a precocious stage of metabolic derangement. This was also confirmed by the direct association of CALR and PDIA3 with the number of metabolic alterations typical of metabolic syndrome. Our findings were in line with similar results in adults with metabolic syndrome (42). Furthermore, CALR expression increased during an OGTT in healthy adults, suggesting that it could be a sensible ER stress marker of chronic and acute dysglycemia in both children and adults (42). Furthermore, a decreased CALR expression in adipose tissue after Roux-en-Y gastric bypass in obese adult patients has been shown, suggesting a link with the decrease in chronic inflammation and metabolic derangement (43).

This study has the limitation of having a small number of samples analyzed, which is secondary to the experimental difficulties faced in the development of the dosage of PDIA3 and CALR since their range for pediatric age is not yet known. Because of the lack of data in the literature that correspond to the results of our study, analysis for the expansion of the sample is needed to confirm our preliminary findings.

In conclusion, this pilot study aimed to establish the association between ER stress and metabolic complications in obese children and adolescents. The results suggest the existence of an important link between ER stress and metabolic changes behind obesity complications even in pediatric age. CALR and PDIA3 could be early markers of insulin resistance and dyslipidemia-related ER stress, being useful to stratify patients at high risk of further complications over time.

## Data availability statement

The original contributions presented in the study are included in the article/Supplementary Material. Further inquiries can be directed to the corresponding authors.

## Ethics statement

Ethical review and approval was not required for the study on human participants in accordance with the local legislation and institutional requirements. Written informed consent to participate in this study was provided by the participants’ legal guardian/next of kin.

## Author contributions

Conceptualization: FP, SB, and MC. Methodology: VA, CP, MG, FP, SB, and MC. Software: DS. Validation: RR and MaC. Formal analysis: VA, DS, and FP. Investigation: VA, RR, VM, and ST. Data curation: MC. Writing—original draft preparation: VA, FGC, and FP. Writing—review and editing: SB and MC. Supervision: SB, MaC, and FP. Funding acquisition: FP, SB, and MC. All authors discussed the results and contributed to the final manuscript. All authors affirm that the present work is original, has not been published previously, and has not been submitted elsewhere for consideration of print or electronic publication. Each person listed as an author participated in the work in a substantive manner, in accordance with ICMJE authorship guidelines, and is prepared to take public responsibility for it.

## Funding

This research was partially supported by a Department of Excellence grant (FOHN project, KE-TOMI project) and PRIN grant (2020NCKXBR_004; SIDERALE Project) from the Ministry of Education, Universities, and Research (MUR), DMPREVENT project and Università del Piemonte Orientale (FAR–2016).

## Acknowledgments

The authors would like to thank all the children and their parents for their contributions to this study.

## Conflict of interest

The authors declare that the research was conducted in the absence of any commercial or financial relationships that could be construed as a potential conflict of interest.

## Publisher’s note

All claims expressed in this article are solely those of the authors and do not necessarily represent those of their affiliated organizations, or those of the publisher, the editors and the reviewers. Any product that may be evaluated in this article, or claim that may be made by its manufacturer, is not guaranteed or endorsed by the publisher.

## References

[1] Chooi YC Ding C Magkos F. The epidemiology of obesity. Metabolism (2019) 92:6–10. doi: 10.1016/j.metabol.2018.09.005 30253139

[2] Bray GA. Medical consequences of obesity. J Clin Endocrinol Metab (2004) 89:2583–9. doi: 10.1210/jc.2004-0535 15181027

[3] Abarca-Gómez L Abdeen ZA Hamid ZA Abu-Rmeileh NM Acosta-Cazares B Acuin C. Trends in body-mass index, underweight, overweight, and obesity from 1975 to 2016: A pooled analysis of 2416 population-based measurement studies in 128·9 million children, adolescents, and adults. Lancet (2017) 390:2627–42. doi: 10.1016/S0140-6736(17)32129-3 PMC5735219 29029897

[4] Afshin A Forouzanfar MH Reitsma MB Sur P Estep K Lee A. Health effects of overweight and obesity in 195 countries over 25 years. N Engl J Med (2017) 377:13–27. doi: 10.1056/nejmoa1614362 28604169 PMC5477817

[5] Longo M Zatterale F Naderi J Parrillo L Formisano P Raciti GA. Adipose tissue dysfunction as determinant of obesity-associated metabolic complications. Int J Mol Sci (2019) 20:2358. doi: 10.3390/ijms20092358 PMC6539070 31085992

[6] Blüher M. Adipose tissue inflammation: a cause or consequence of obesity-related insulin resistance? Clin Sci (2016) 130:1603–14. doi: 10.1042/CS20160005 27503945

[7] Trayhurn P Wang B Wood IS. Hypoxia in adipose tissue: A basis for the dysregulation of tissue function in obesity? British J Nutr (2008) 100:227–35. doi: 10.1017/S0007114508971282 18397542

[8] Singh P Hoffmann M Wolk R Shamsuzzaman ASM Somers VK. Leptin induces c-reactive protein expression in vascular endothelial cells. Arterioscler Thromb Vasc Biol (2007) 27(9):e302-7. doi: 10.1161/ATVBAHA.107.148353 17615382

[9] Savini I Catani MV Evangelista D Gasperi V Avigliano L. Obesity-associated oxidative stress: Strategies finalized to improve redox state. Int J Mol Sci (2013) 14:10497–538. doi: 10.3390/ijms140510497 PMC3676851 23698776

[10] Lyons CL Kennedy EB Roche HM. Metabolic inflammation-differential modulation by dietary constituents. Nutrients (2016) 8:247. doi: 10.3390/nu8050247 PMC4882660 27128935

[11] Schröder M. Endoplasmic reticulum stress responses. Cell Mol Life Sci (2008) 65:862–94. doi: 10.1007/s00018-007-7383-5 PMC11131897 18038217

[12] Hotamisligil GS. Endoplasmic reticulum stress and the inflammatory basis of metabolic disease. Cell (2010) 140:900–17. doi: 10.1016/j.cell.2010.02.034 PMC2887297 20303879

[13] Boden G Duan X Homko C Molina EJ Song W Perez O. Increase in endoplasmic reticulum stress–related proteins and genes in adipose tissue of obese, insulin-resistant individuals. Diabetes (2008) 57:2438–44. doi: 10.2337/db08-0604 PMC2518495 18567819

[14] Caira S Iannelli A Sciarrillo R Picariello G Renzone G Scaloni A. Differential representation of liver proteins in obese human subjects suggests novel biomarkers and promising targets for drug development in obesity. J Enzyme In-hib. Med Chem (2017) 32:672–82. doi: 10.1080/14756366.2017.1292262 PMC6009959 28274171

[15] Murri M Insenser M Bernal-Lopez MR Perez-Martinez P Escobar-Morreale HF Tinahones FJ. Proteomic analysis of visceral adipose tissue in pre-obese patients with type 2 diabetes. Mol Cell Endocrinol (2013) 376:99–106. doi: 10.1016/j.mce.2013.06.010 23791845

[16] Alfadda AA Benabdelkamel H Masood A Moustafa A Sallam R Bassas A. Proteomic analysis of mature adipocytes from obese patients in relation to aging. Exp Gerontol. (2013) 48:1196–203. doi: 10.1016/j.exger.2013.07.008 23886751

[17] Trombetta ES Helenius A. Conformational requirements for glycoprotein reglucosylation in the endoplasmic reticulum. J Cell Biol (2000) 148:1123–30. doi: 10.1083/jcb.148.6.1123 PMC2174309 10725325

[18] Turano C Gaucci E Grillo C Chichiarelli S. PDIA3/GRP58: A protein with multiple functions. Cell Mol Biol Lett (2011) 16:539–63. doi: 10.2478/s11658-011-0022-z PMC6275603 21837552

[19] Schröder M Kaufman RJ. The mammalian unfolded protein response. Annu Rev Biochem (2005) 74:739–89. doi: 10.1146/annurev.biochem.73.011303.074134 15952902

[20] Calfon M Zeng H Urano F Till JH Hubbard SR Harding HP. IRE1 couples endoplasmic reticulum load to secretory capacity by processing the XBP-1 mRNA. Nature (2002) 415:92–6. doi: 10.1038/415092a 11780124

[21] Boden G Lebed B Schatz M Homko C Lemieux S. Effects of acute changes of plasma free fatty acids on intra-myocellular fat content and insulin resistance in healthy subjects. Diabetes (2001) 50:1612–7. doi: 10.2337/diabetes.50.7.1612 11423483

[22] Silvennoinen L Myllyharju J Ruoppolo M Orrù S Caterino M Kivirikko KI. Identification and characterization of structural domains of human PDIA3: Association with calreticulin requires several domains. J Biol Chem (2004) 279:13607–15. doi: 10.1074/jbc.M313054200 14732712

[23] Cole TJ Lobstein T. Extended international (IOTF) body mass index cut-offs for thinness, overweight and obesity. Pediatr Obes (2012) 7:284–94. doi: 10.1111/j.2047-6310.2012.00064.x 22715120

[24] Tanner JM. Growth at adolescence. with a general consideration of the effects and environmental factors upon growth and maturation from birth to maturity; 2d ed. Oxford: Blackwell Scientific Publications (1962).

[25] Flynn JT Kaelber DC Baker-Smith CM Blowey D Carroll AE Daniels SR. Clinical practice guideline for screening and management of high blood pressure in children and adolescents. Pediatrics (2017) 140(3):e20171904. doi: 10.1542/peds.2017-1904 28827377

[26] Cruz ML Goran MI. The metabolic syndrome in children and adolescents. Curr Diab. Rep (2004) 4:53–62. doi: 10.1007/s11892-004-0012-x 14764281

[27] Ricotti R Solito A Zani EM Caputo M Genoni G Barone-Adesi F. The relationship between cortisol and IGF-I influences metabolic alteration in pediatric overweight and obesity. Eur J Endocrinol (2020) 182:255–64. doi: 10.1530/EJE-19-0792 31863690

[28] Genoni G Menegon V Monzani A Archero F Tagliaferri F Mancioppi V. Healthy lifestyle intervention and weight loss improve cardiovascular dysfunction in children with obesity. Nutrients (2021) 13:130. doi: 10.3390/nu13041301 PMC8071179 33920831

[29] Raghavan M Wijeyesakere SJ Peters LR Del Cid N. Calreticulin in the immune system: Ins and outs. Trends Immunol (2013) 34:13–21. doi: 10.1016/j.it.2012.08.002 22959412 PMC4117402

[30] Charonis AS Michalak M Groenendyk J Agellon LB. Endoplasmic reticulum in health and disease: The 12th interna-tional calreticulin workshop, Delphi, Greece. J Cell Mol Med (2017) 21:3141–9. doi: 10.1111/jcmm.13413 PMC5706586 29160038

[31] Kageyama S Isono T Iwaki H Wakabayashi Y Okada Y Kontani K. Identification by proteomic analysis of calreticulin as a marker for bladder cancer and evaluation of the diagnostic accuracy of its detection in urine. Clin Chem (2004) 50:857–66. doi: 10.1373/clinchem.2003.027425 14764641

[32] Leys CM Nomura S LaFleur BJ Ferrone S Kaminishi M Montgomery E. Expression and prognostic significance of prothymosin-α and PDIA3 in human gastric cancer. Surgery (2007) 141:41–50. doi: 10.1016/j.surg.2006.05.009 17188166

[33] Linz A Knieper Y Gronau T Hansen U Aszodi A Garbi N. ER stress during the pubertal growth spurt results in impaired long-bone growth in chondrocyte-specific PDIA3 knockout mice. J Bone Miner. Res (2015) 30:1481–93. doi: 10.1002/jbmr.2484 25704664

[34] Bravo R Parra V Gatica D Rodriguez AE Torrealba N. Endoplasmic reticulum and the unfolded protein response. Int Rev Cell Mol Biol (2013) 301:215–90. doi: 10.1016/B978-0-12-407704-1.00005-1 PMC3666557 23317820

[35] Linnik KM Herscovitz H. Multiple molecular chaperones interact with apolipoprotein b during its maturation: The network of endoplasmic reticulum-resident chaperones (ERp72, GRP94, calreticulin, and BiP) interacts with apolipoprotein b regardless of its lipidation state. J Biol Chem (1998) 273:21368–73. doi: 10.1074/jbc.273.33.21368 9694898

[36] Stillemark P Borén J Andersson M Larsson T Rustaeus S Karlsson KA. The assembly and secretion of apolipoprotein b-48-containing very low-density lipoproteins in McA-RH7777 cells. J Biol Chem (2000) 275:10506–13. doi: 10.1074/jbc.275.14.10506 10744742

[37] Maffeis C. L’obesità̀ del bambino: Aspetti clinici e fisiopatologici. Torino: Centro Scientifico editore (2009).

[38] Ahrens W Pigeot I Pohlabeln H De Henauw S Lissner L Molnár D. Prevalence of overweight and obesity in European children below the age of 10. Int J Obes (2014) 38:S99–S107. doi: 10.1038/ijo.2014.140 25376223

[39] Özcan U Cao Q Yilmaz E Lee AH Iwakoshi NN Özdelen E. Endoplasmic reticulum stress links obesity, insulin action, and type 2 diabetes. Sci (80-.) (2004) 306:457–61. doi: 10.1126/science.1103160 15486293

[40] Czaja MJ. JNK regulation of hepatic manifestations of the metabolic syndrome. Trends Endocrinol Metab (2010) 21:707–13. doi: 10.1016/j.tem.2010.08.010 PMC2991513 20888782

[41] Jalali S Aghasi M Yeganeh B Mesaeli N. Calreticulin regulates insulin receptor expression and its downstream PI3 ki-nase/Akt signalling pathway. Biochim Biophys Acta - Mol Cell Res (2008) 1783:2344–51. doi: 10.1016/j.bbamcr.2008.08.014 18840478

[42] Sage AT Holtby-Ottenhof S Shi Y Damjanovic S Sharma AM Werstuck GH. Metabolic syndrome and acute hyperglycemia are associated with endoplasmic reticulum stress in human mononuclear cells. Obesity (2012) 20:748–55. doi: 10.1038/oby.2011.144 21633399

[43] Ferraz-Bannitz R Welendorf CR Coelho PO Salgado W Jr Nonino CB Beraldo RA. Bariatric surgery can acutely modulate ER-stress and inflammation on subcutaneous adipose tissue in non-diabetic patients with obesity. Diabetol Metab Syndr (2021) 13(1):19. doi: 10.1186/s13098-021-00623-w 33593418 PMC7887793

